# FDA Over‐The‐Counter Hearing Aid (OTC HA) Rule guides innovation to assist those with risk of cognitive decline

**DOI:** 10.1002/alz.087311

**Published:** 2025-01-09

**Authors:** Elora Gupta

**Affiliations:** ^1^ Audition Technology, Pittsburgh, PA USA

## Abstract

The recent ACHIEVE study (https://www.achievestudy.org/) demonstrated the substantial benefit of hearing aid use in those with mild‐moderate hearing loss and at increased risk for cognitive decline. The FDA’s OTC HA Rule provides a reliable basis for technology innovation to improve accessibility, affordability and wear of quality hearing aids in this population.

For over 3 years, we have been collecting real‐world data on user needs from individuals with cognitive decline risks such as age, family history, prior brain injury, heart disease and diabetes, mental health concerns, employment in high noise hazard and fall hazard industries. These individuals also had diagnosed or self‐perceived hearing loss, one‐sided hearing loss, hyperacusis, tinnitus, balance problems. We sought input on (1) OTC HA expectations (2) meaningful product features and tools (3) willingness to try OTC HAs.

The key needs for purchase decision‐making and consistent wear were:

1. *Education on user‐centricity*: Understanding how the FDA effectiveness and safety requirements translate to daily lifestyle and individual hearing needs

2. *Confidence in quality*: Understanding scope of FDA’s technical and labeling requirements and ability to distinguish from misbranded products

3. *Design supporting daily activities and lifestyle*: Ease of use with limitations such as avoiding social engagement and healthcare appointments, remembering HA settings, judging loudness of environmental sounds, losing hearing aids, restricted mobility and dexterity, other hearing problems such as tinnitus

4. *Caregiver engagement to improve hearing aid wear*: Joint decision‐making for OTC purchase, setting realistic expectations, monitoring and supporting wear, detecting worsening in hearing abilities or red flags requiring ENT consult

5. *Tools to protect remaining hearing*: From noise, ototoxins, earwax, other risk factors and comorbidities

6. *Improving overall health and quality of life*: Reliable tools for monitoring and potentially improving physical and mental health, in conjunction with hearing health, individualized and self‐paced learning that is interactive with privacy protection

7. *Cost‐effectiveness and convenience*: Fair pricing with quality assurance per FDA requirements, payment flexibility, maintenance at home, communicating with manufacturer

These needs can be integrated into OTC HA design and functionalities, with tools to specifically benefit those with mild‐moderate hearing loss and cognitive decline risks.

